# Transformation of NSCLC to SCLC after 1st- and 3rd-generation EGFR-TKI resistance and response to EP regimen and erlotinib: 2 CARE-compliant case reports

**DOI:** 10.1097/MD.0000000000025046

**Published:** 2021-03-12

**Authors:** Lin Lai, Wentao Meng, Jialiang Wei, Xiaofei Zhang, Zhiwei Tan, Yunxin Lu, Encun Hou

**Affiliations:** Ruikang Hospital Affiliated to Guangxi University of Chinese Medicine, Nanning, Guangxi, People's Republic of China.

**Keywords:** case report, EGFR-TKI resistance, erlotinib, etoposide/cisplatin, non-small-cell lung cancer, small-cell lung cancer transformation

## Abstract

**Rationale:**

Genotypic and histological evolution of non-small-cell lung cancer (NSCLC) into small-cell lung cancer (SCLC) has been described as a mechanism of acquired resistance to epidermal growth factor receptor (EGFR) tyrosine kinase inhibitor (TKI) therapy. However, the number of clinical cases is rare.

**Patient concerns:**

Two lung adenocarcinoma patients with EGFR mutations who recurred after radical resection transformed into SCLC under treatment with the sequential first- and third-generation EGFR-TKIs.

**Diagnosis:**

The 2 cases were both confirmed as SCLC by pathological rebiopsy after EGFR-TKIs resistance.

**Interventions:**

Case 1 was treated with etoposide plus cisplatin (EP) regimen and erlotinib, while case 2 was treated with erlotinib and EP followed by oral etoposide.

**Outcomes:**

Case 1 treated with EP only achieved 3-month progression-free survival (PFS), which is the first case that reported T790 M/C797S cis-mutation for osimertinib resistance before the SCLC transformation. However, case 2 treated with erlotinib and EP followed by oral etoposide, PFS lasted for 8 months.

**Lessons:**

The cases highlighted the importance of rebiopsy that identified pathologically SCLC transformation after EGFR-TKI resistance, and suggested the treatment of erlotinib plus EP followed by etoposide, which could provide a reference for such phenotype.

## 1 Introduction

EGFR-directed TKIs play very important roles in treatment of advanced epidermal growth factor receptor (EGFR) mutated non-small-cell lung cancer (NSCLC). However, acquired resistance may develop inevitably. T790 M mutation accounts for approximately 60% of the resistance cases in first-generation tyrosine kinase inhibitor (TKIs) treatment.^[1,2]^ Third-generation TKIs such as osimertinib were effective against T790 M mutated NSCLC, with overall response rate of about 60%, but acquired resistance occur in about 10 months.^[3]^ After either first-generation or third-generation EGFR TKIs, transformation to SCLC has been described as a mechanism of resistance to EGFR TKIs in approximately 5% of patients.^[1,2,4]^

Here we reported 2 patients with advanced EGFR mutated lung adenocarcinomas treated with the first-generation TKI and the subsequent osimertinib. Both of these 2 patients then transformed into EGFR TKI-resistant SCLCs retaining the EGFR exon 19 deletion. But the patient treated with erlotinib plus EP followed by oral etoposide achieved longer progression-free survival (PFS) than the 1 treated with etoposide and cisplatin (EP) therapy since small-cell lung cancer (SCLC) transformation. Two case reports were approved by the Ethics Committee of Ruikang Hospital Affiliated to Guangxi University of Chinese Medicine. Written informed consents were obtained from the parents or spouses of all participants for publication of their case reports and accompanying images.

## 2 Case presentation

### 2.1 Case 1

**Figure 1:**
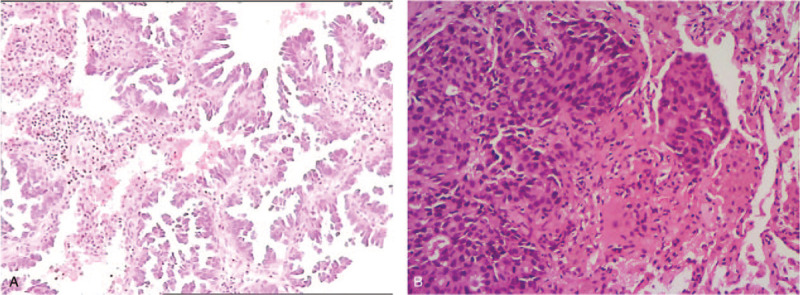
Surgical samples of lung cancer showed histopathology of adenocarcinoma (Hematoxylin-eosin staining, 200 X). (A) Tissue section from case 1. (B) Tissue section from case 2.

**Figure 2:**
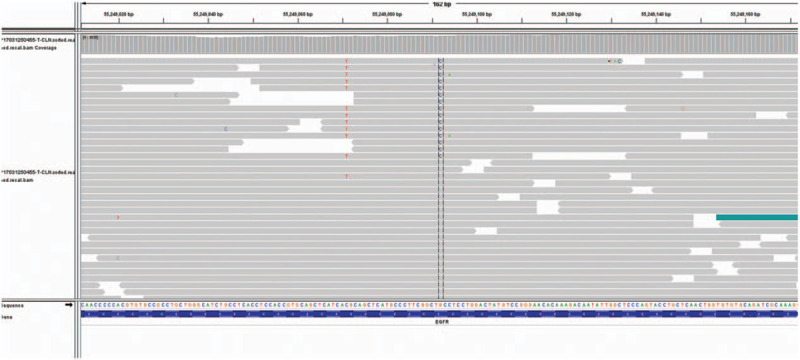
In case 1, a next-generation sequence showed EGFR T790 M/C797S cis-mutation after osimertinib treatment.

**Figure 3:**
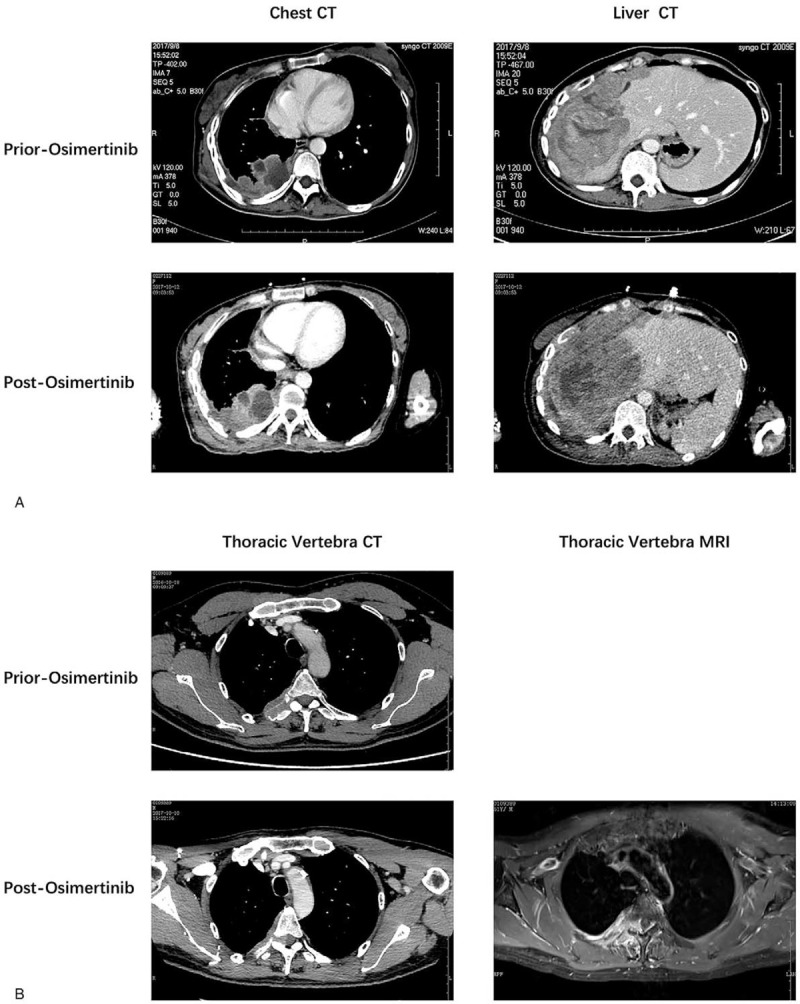
Computed tomography (CT) and magnetic resonance imaging (MRI) images of prior and postosimertinib treatment showed disease progress. (A) Case 1. (B) Case 2.

**Figure 4:**
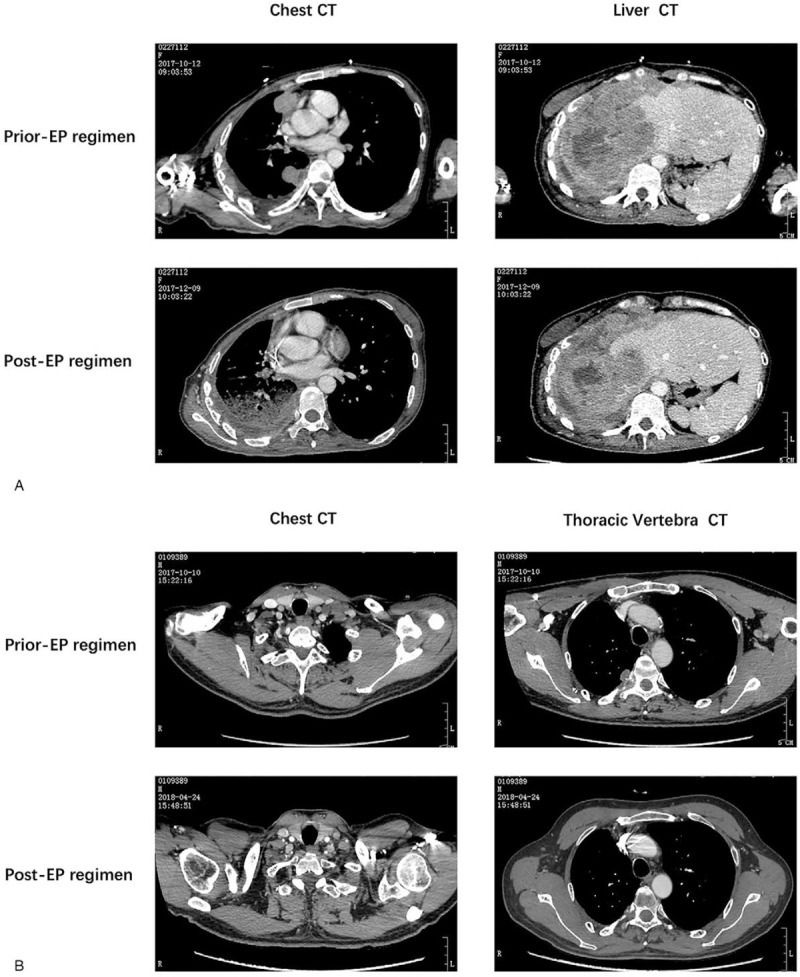
Rebiopsy samples after osimertinib resistance showed transformation to small cell lung cancer (Hematoxylin-eosin staining, 200 X). (A) Tissue section from Case 1. (B) Tissue section from Case 2.

**Figure 5:**
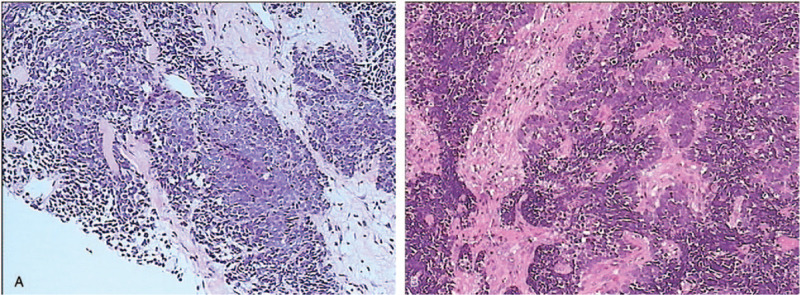
Computed tomography (CT) images of prior and postEP treatment showed therapeutic response. (A) Case 1. (B) Case 2.

In December 2013, a 34-year-old non-smoker female was diagnosed with right lung adenocarcinoma (pT3N2M0, stage IIIA) (Fig. 1A) harboring an EGFR exon 19 mutation and underwent curative surgery. Four cycles of adjuvant chemotherapy with pemetrexed and cisplatin regimen (pemetrexed, 500 mg/m^2^ plus cisplatin 75 mg/m^2^) were administered after surgery. In August 2014, new lesions were found in her right pleura and brain (cT3N2M1b, IVA), so the patient underwent one-cycle gemcitabine and cisplatin regimen (gemcitabine, 1000 mg/m^2^ plus cisplatin 75 mg/m^2^) and whole brain radiation therapy. Then the patient was given oral erlotinib (150 mg per day) until the lesions progressed in February 2016. A plasma circulating tumor DNA (ctDNA) biopsy showed a T790 M missense mutation in EGFR exon 20 accompanied by exon 19 deletion. So she switched to osimertinib (80 mg per day) from Febuary 2016 to Febuary 2017. And then, due to the chest pain, next generation sequencing of ctDNA was performed which showed EGFR exon 19 deletion, T790 M and C797S cis-mutations (Fig. 2), RB1 c.343_380+4del and TP53 p.Q144X nonsense mutation. Her symptoms, especially the pain in right chest and upper abdomen, gradually aggravated in the next 5 months and she achieved progressed disease in July 2017. Then the patient was given chemotherapy with pemetrexed (500 mg/m^2^) plus bevacizumab (15 mg/kg). But disease progressed after 2 cycles (Fig. 3A). In October 2017, the punctured biopsy of right chest wall histologically showed SCLC (Fig. 5A) and the tissue biopsy showed loss of T790 M and C797S mutations but remained exon 19 del. From October 2017, the patient received EP (etoposide, 100 mg/m^2^ plus cisplatin 75 mg/m^2^) combination therapy and orally erlotinib with a dose of 150 mg once a day and achieved a stable disease (Fig. 4A). Unfortunately, the patient passed away because of acute pulmonary embolism in January 2018.

### 2.2 Case 2

In April 2014, a 48-year-old male, with a smoking history for more than 30 years with 20 pigs/day, received curative surgery in right lung. The postoperative pathology showed lung adenocarcinoma (pT2aN1M0, stage IIA) (Fig. 1B) with EGFR exon 19 deletion. He received 4-cycles of postoperative pemetrexed and cisplatin chemotherapy (pemetrexed, 500 mg/m^2^ plus cisplatin 75 mg/m^2^). In December 2014, local regional recurrence and contralateral lobe metastases were observed (cT2aN3M1a, IVA), so the patient was treated with orally administered gefitinib with a dose of 250 mg, once a day. In 2016, because of bony pain of thoracic vertebrae and gefitinib resistance, the ctDNA test was performed which showed T790 M mutation. So the patient was given osimertinib (80 mg, once a day). In October 2017, due to aggravation of thoracic vertebrae destruction and paraplegia (Fig. 3B), the patient received left supraclavicular lymph node biopsy which pathologically showed SCLC (Fig. 5B). Genetic testing suggested EGFR exon 19 del with RB1 exon 7_exon17 del and TP53 exon 8 nonsense mutation. In November 2017, the patients underwent stereotactic body radiation therapy (SBRT) for metastasis of spinal cord and 6 cycles of EP (etoposide, 100 mg/m^2^ plus cisplatin 75 mg/m^2^) therapy and sequential 2 cycles of oral etoposide mono-therapy (100 mg/m^2^, day1–5, 21 days per cycle), in combination with erlotinib (150 mg per day). The clinical outcome is partial response and symptomatic relief (Fig. 4B). In July 2018, the disease progressed and the patient underwent anlotinib with a dose of 12 mg per day. Unfortunately, the patient passed away due to severe pneumonia in August 2018.

## 3 Discussion

SCLC transformation has been described as a resistance mechanism to EGFR directed TKIs in approximately 5% of patients and has been observed after both first-generation and subsequent third-generation EGFR TKIs.^[1,2,5]^ The 2 cases in this study were initially diagnosed with lung adenocarcinoma harboring EGFR exon 19 mutation. After curative surgeries and adjuvant chemotherapy, owing to recurrence and metastasis, these 2patients were treated with erlotinib or gefitinib, the first-generation EGFR TKIs, respectively. Despite the initial efficacy, the 2 patients developed resistance 1 year later. They both performed the ctDNA genetic assay showing T790 M mutation which is the secondary EGFR mutation occurring in 50% to 65% of resistant rebiopsies.^[6]^ So the 2 patients were treated with osimertinib which is a third-generation EGFR-TKI targeting both T790 M and EGFR-TKI sensitive mutations. osimertinib is very effective but unfortunately acquired resistance occurred in these 2 cases. For case 1, genetic assays showed EGFR exon 19 deletion with T790 M/C797S cis-mutations firstly, and then after 2 cycles of pemetrexed plus bevacizumab, EGFR exon 19 deletion only for SCLC transformation. For case 2, after osimertinib resistance, the genetic assay showed EGFR exon 19 deletion accompanied with SCLC transformation. After small cell lung cancer transformation, the EGFR exon19 mutations were retained, suggesting that they may be derived from the same origin as the original lung adenocarcinoma, whereas the T790 M mutation lost after transformation, which was consistent with the literature.^[7]^

Acquired C797S mutation was thought to be one of the resistance mechanisms associated with osimertinib treatment.^[8,9]^ Notably, in case 1 the ctDNA assay suggested T790 M/C797S cis-mutation for osimertinib resistance before the SCLC transformation. To our knowledge, this is the first case that reported SCLC transformation after T790 M/C797S cis-mutation.

For case 1, overall survival since diagnosis was 49 months, whereas survival time since SCLC transformation was 3 months. For case 2, overall survival since diagnosis was 52 months, whereas survival time since SCLC transformation was 10 months while PFS lasted for 8 months. The enormous difference of survival time since SCLC transformation between these 2 patients might due to the combination therapy of erlotinib with EP followed by etoposide. To our knowledge, this is the first case that reported about the efficacy of combination therapy of erlotinib with EP followed by etoposide on SCLC transformation patients with EGFR exon19 deletion.

In summary, we reported 2 cases of SCLC transformation during treatment with osimertinib. Genomic study showed that these transformed SCLCs are characterized loss of the T790 M mutation in the presence of the EGFR exon 19 deletion. Currently, patients with NSCLC transformed into SCLC after EGFR-TKI resistance could continue EGFR-TKI and combine with the standard chemotherapy regimen of SCLC. However, the optimal regimen needs to be further confirmed.^[10]^

## 4 Conclusions

These cases suggest that rebiopsy is essential for patients suffered from disease progresses after EGFR-TKI resistance, so treatment can be adjusted according to the diverse mechanisms of acquired resistance. More importantly, comparison of survival time since SCLC transformation between these 2 patients revealed that erlotinib with EP followed by oral etoposide might benefit patient of SCLC transformation harboring EGFR exon19 deletion.

## Author contributions

**Conceptualization:** Yunxin Lu, Encun Hou.

**Data curation:** Lin Lai, Wentao Meng.

**Investigation:** Jialiang Wei, Xiaofei Zhang, Zhiwei Tan.

**Methodology:** Jialiang Wei, Xiaofei Zhang, Zhiwei Tan.

**Writing – original draft:** Lin Lai.

**Writing – review & editing:** Lin Lai, Yunxin Lu, Encun Hou.
